# Some Observation on the Treatment of Delayed Union of Bones

**Published:** 1911-10

**Authors:** L. Sexton

**Affiliations:** Minor Surgery, Tulane University


THE
### TEXAS MEDICAL JOURNAL.
Established July, 1885

F. E. DANIEL, M. D., - - - - - Editor, Publisher and Proprietor

Published Monthly.—Subscription $1.00 a Year

VOL. XXVII. AUSTIN, OCTOBER, 1911. No. 4.

The publisher is not responsible for the views of the contributors.

### Original Articles.

For Texas Medical Journal.

### Some Observation on the Treatment of Delayed Union of Bones.

BY L. SEXTON, B. S., M. D., MINOR SURGERY, TULANE UNIVERSITY.

One per cent of fractures of the long bones, patella and olecranon process are united by faulty, delayed, ligamentous or non-union. As the better teaching method of the treatment of fractures begins to bear fruit, and the compound fractures are properly approximated by Lane plates or sutures, this percentage of faulty unions will be reduced and better general results shown in treating fractures.

*Causes.*—Many diseases, such as general debility, osteomalacia, scurvy, rickets, syphilis and tuberculosis, are the constitutional causes of delayed union. The local causes may be defective innervation, osteomyelitis, lack of blood supply, muscles, tendons or soft parts between the fractured bones, defective apposition from muscular action and splints which do not hold the two ends of bone in apposition, interposition of fluids and aponeurotic tissue, detached fragments of bone, want of rest (by not fixing the joint above and below the fracture), damage to the nutrient artery (as in fracture of the cervical neck of the femur), malignant tumors of the bone, extensive injury to the periosteum, inflammation and alcoholism. While these are the more frequent causes of non-union, there may be other conditions from general cachexia and blood deficiencies that also tend to prevent the union of bone.

When the above causes of non-union of bones have resulted in the femur, tibia and bones of the upper and lower arm not becoming firmly united, there was a certain period of time in which many surgeons advocate various methods outside of actual resection of the false joint. Some of the methods applied might cause hardening or osteoblasts to be developed where we had fibrous union, but in actual cases of non-union there is no method practically outside of resecting the ends of the bone. X-ray plates and the fluoroscope ought to aid us in distinguishing as to which particular case requires pegs or irritation and the other cases which necessitate actual resection.

*Symptoms.*—Preternatural mobility without crepitus where the bone should be firm, as a false joint in the femur or humerus, a deep sulci where the surface should be smooth, instanced in fracture of the patella with the fragments drawn apart. Flail arm or leg with loss of function are some of the symptoms of delayed union of bones.

*Varieties.*—First. In some cases there is no reparative process started, as in fracture of a bone affected by sarcoma.

Second. A ligamentous union by a mass of connective and fibrous tissue. In this variety the osteoblasts do not enter into fibrous tissue in order to harden the same, leaving a flail limb, which for service is hardly any better than where absolute non-union has been the result.

Third. Where a false joint in which the ends of the fractured bone are covered by cartilaginous tissue, which has no tendency to unite. The constant friction between the ends of such a fracture produces a sort of ball and socket joint, so it has been designated pseudarthrosis.

The most common site for ununited fractures are the lower third of the femur, the middle shaft of the humerus, the coracoid and olecranon process and the patella, the latter on account of the strong muscles and ligaments attached to it.

*Prognosis.*—The prognosis depends upon the age, cause and treatment of the condition. The cause of delayed union in children usually is some cachexia, or disease of the bone. Prognosis is not so favorable, as the bones may become atrophied or pointed, covered with cartilaginous tissue, in which there is no prospect of union. These are the varieties of cases that after repeated operative procedures have failed to unite, finally necessitating the amputation of the limb.

*Treatment.*—Constitutional treatment consists in a change of climate and environments when not suitable to the health of the patient, the giving of tonic, such as lime-salts, strychnea, arsenic and quinine. The local method of treatment is the rubbing of the two ends of bone rather harshly together, followed by the fixing of the limb or extremity in a well-padded plaster of Paris dressing, which is kept on for six weeks or two months, and may be reapplied at the end of this time, provided there is no evidence of healing.

The ambulatory way of treating ununited fractures of the leg is by massage, keeping up and walking about in the fresh air, leaving the limb to hang down, producing an increased blood supply to the part, or applying a rubber bandage around the limb for an hour or two daily, as in the Bier method of treatment. Failing in these paliative measures, there is nothing left to be done but the open method or operative treatment. When the bones are not covered with thick muscles and the deformity is not too great, the ends may be freshened by a Giglie saw or bone pliers, fitted and secured together by silver wire or kangaroo tendon, both of which is to be aseptically applied. Another newer method of holding the bones together is by the application of Lane plates, consisting of a narrow piece of metal with four screw holes, two on either side of the fracture, which are firmly applied so as to approximate and hold the two fractured ends in position.

To properly apply and fit these plates where the muscles are large and the bones are deeply covered necessitates an extensive operation which should not be undertaken by a novice in surgery. Another method of treatment is by boring holes into each fragment into which ivory pegs are driven. The pegs may either be removed or remain permanently. They are the nucleus for a large amount of new bone material by which the fracture is finally firmly united.

Delayed union, as said before, is a slow recuperative process, where the osteoblasts are retarded in ossifying the callus. There is a method of stimulating this process by increasing local blood supply and by rubbing the ends of the bone together. Some cases require pegs or irritation and still other cases which necessitate actual wiring or suturing. Strict antisepsis, a large enough incision to deliver the ends of the bone is required in the latter class. Cut or saw the ends squarely across so that co-aptation can be well made with Lane's plates or silver wire, placing the two ends of the resected bone in immediate juxtaposition when the operation is finished and the splints applied.

As a matter of course, incision should be made on the opposite side to the blood vessels and where the bone is nearest the surface. The ends of the bones may be amputated by a Giglie saw, which, however, is liable to break, or by sharp chisel and mallet or bone pliers.

As stated before, the cut across the bone should be exactly transverse, as the future union of the bones depends largely upon this one point. The case is then treated as in other compound fractures, with immobilization the chief object to be obtained. We must always remember in using silver wire that absorption of the bone around it is likely to take place. The principal object in using the wire is that with it you can overcome lateral displacement. The other advantage about the wire is that no sepsis can come from that kind of suture. Wire and staples act as foreign bodies, may cause late suppuration, and require a second operation to remove them. Many surgeons fearing the results incident to this criticism rely largely upon kangaroo tendon and chromacized catgut to hold the ends of the fragments in position. Some surgeons who have had experience prefer to approximate and hold the two fragments together by twenty-day chromacized catgut or kangaroo tendon on account of the absorbability of this suture material. If they hold a bone together for ten days, or two weeks, enough callus has developed to unite the fractures, and in very many cases in which the pegs, staples, Lane plates, or wire sutures have been applied to approximate the parts, they later fail to become either encysted or absorbed, acting as foreign bodies having to be removed at some future time, thus necessitating a second operation. Another plan has been to bore holes into the ununited callus and into the bone.

After any of these methods of treatment, immobilize the part five or six months, if good union does not take place earlier.

After the bones have been thoroughly approximated and the splints and dressing applied, if convenient, an X-ray plate should be taken in order to show that the bones have not been broken apart in the manipulation incident to applying the outer dressings. It is strange to say, but, nevertheless, true, that in occasional cases of fracture which have been at one time firmly united, the callus either absorbs or separates, producing a condition known as disunited fracture. This is only likely to occur in cases in which there is some wasting disease, such as scurvy, tuberculosis or lues.

With the wire suture we have recently had a successful termination of two cases of ununited fracture, one being in the humerus and the other in the femur. In each case we cut down upon the false joint on the opposite side to the nerves and blood-vessels, freshened the ends of the bone by a Giglie saw and bone nippers, drilled two holes through the shafts on either side of the fracture and approximated the ends, twisting the wire together.

In the femur case we had a slight infection from the flesh wound, as it was treated in a ward with numerous other pus cases, but the patient made an uneventful recovery, though he had worn out half a dozen plaster of Paris casts, and had been confined in the hospital for more than seven months. In the fracture of the humerus we managed to miss infection, had no rise of temperature, though there was considerable traumatism, the bones united perfectly, as shown by X-ray plates, and in each instance useful limbs were made from previously useless appendages on account of the non-union of the fracture.

